# Assessing *in-vitro* models for microglial development and fetal programming: a critical review

**DOI:** 10.3389/fimmu.2025.1538920

**Published:** 2025-01-29

**Authors:** Steven Schepanski, Gonza B. Ngoumou, Claudia Buss, Georg Seifert

**Affiliations:** ^1^ Charité - Universitätsmedizin Berlin, corporate member of Freie Universität Berlin and Humboldt-Universität zu Berlin, Charité Competence Center for Traditional and Integrative Medicine (CCCTIM), Berlin, Germany; ^2^ Charité - Universitätsmedizin Berlin, corporate member of Freie Universität Berlin and Humboldt-Universität zu Berlin, Department of Pediatrics, Division of Oncology and Hematology, Berlin, Germany; ^3^ Charité - Universitätsmedizin Berlin, Corporate Member of Freie Universität Berlin, Humboldt-Universität zu Berlin, Institute of Medical Psychology, Berlin, Germany; ^4^ University of California, Irvine, Development, Health and Disease Research Program, Irvine, CA, United States; ^5^ German Center for Child and Adolescent Health (DZKJ), Partner Site Berlin, Charité - Universitätsmedizin Berlin, Berlin, Germany; ^6^ German Center for Mental Health (DZPG), Partner Site Berlin, Charité - Universitätsmedizin Berlin, Berlin, Germany

**Keywords:** microglia, brain development, *in-vitro* models, neuroimmunology and microglia, neurodevelopmental disorders, neuropsychiatry, developmental origin of health and diseases, fetal programming

## Abstract

This review evaluates *in-vitro* models for studying how maternal influences during pregnancy impact the development of offspring microglia, the immune cells of the central nervous system. The models examined include primary microglia cultures, microglia cell lines, iPSC-derived microglia, PBMC-induced microglia-like cells, 3D brain organoids derived from iPSCs, and Hofbauer cells. Each model is assessed for its ability to replicate the *in-vivo* environment of the developing brain, with a focus on their strengths, limitations, and practical challenges. Key factors such as scalability, genetic and epigenetic fidelity, and physiological relevance are highlighted. Microglia cell lines are highly scalable but lack genetic and epigenetic fidelity. iPSC-derived microglia provide moderate physiological relevance and patient-specific genetic insights but face operational and epigenetic challenges inherent to reprogramming. 3D brain organoids, derived from iPSCs, offer an advanced platform for studying complex neurodevelopmental processes but require extensive resources and technical expertise. Hofbauer cells, which are fetal macrophages located in the placenta and share a common developmental origin with microglia, are uniquely exposed to prenatal maternal factors and, depending on fetal barrier maturation, exhibit variable epigenetic fidelity. This makes them particularly useful for exploring the impact of maternal influences on fetal programming of microglial development. The review concludes that no single model comprehensively captures all aspects of maternal influences on microglial development, but it offers guidance on selecting the most appropriate model based on specific research objectives and experimental constraints.

## 1 Introduction

The concept of fetal programming refers to the idea that changes within the intrauterine environment can contribute to the offspring’s vulnerability to developing diseases later in life by shaping their responses to future internal and external stimuli (1, 2). Various maternal challenges such as stress (3, 4), infection (5, 6), and exposure to environmental pollutants (7, 8) can alter gestational biology and thereby reshaping the intrauterine environment. These alterations, especially during critical windows of fetal development, have been associated with an increased risk of neurodevelopmental disorders and other long-term health consequences in the offspring (9–11).

Microglia, the resident macrophages of the central nervous system (CNS), have garnered increasing interest as key mediators of neurodevelopmental processes during these sensitive periods in the womb and afterwards. In the developing brain, microglia exhibit a high degree of responsiveness to extracellular stimuli (12, 13), which enables them to shape neural circuits through mechanisms such as synaptic pruning (14), clearing of apoptotic cells (15–17), and supporting neurogenesis (18). Originating from yolk sac progenitor cells, microglial cells colonize the CNS as early as the fourth week of gestation (9, 19, 20) and continue to perform essential functions throughout life. In the mature brain, microglia are maintained through self-renewal over the entire lifespan (21) and are essential for homeostasis, neuroplasticity (22), and responses to pathogens (23). Beyond that, microglia are implicated in cognitive functions (24, 25), underscoring their dual role in supporting healthy brain function and contributing to neuropathology when dysregulated.

Research, primary from animal models, has demonstrated that maternal-derived factors, such as elevated maternal glucocorticoids (26, 27), cytokines (28, 29) or immune cells (30), and exposure to environmental pollutants such as diesel exhaust particles (31, 32), can interfere with fetal microglial development. These maternal factors can affect fetal microglia development either through being directly transferred across the placenta or by triggering placental responses that alter the intrauterine environment. Such environmental changes have been associated with dysregulated microglial reactivity, manifesting as either heightened sensitivity with excessive synaptic pruning or diminished responsiveness resulting in less pruning (31, 33–36). However, recent evidence suggests that microglia may not be universally required for experience-dependent neural circuit maturation (37). Given microglia’s central role in neural circuit formation, disruptions in microglia function due to these prenatal maternal factors have been associated with an increased risk of neurodevelopmental disorders in the offspring.

While findings from animal models have substantially expanded our understanding of how intrauterine conditions shape fetal microglial development, translating these findings to humans remains challenging (38). Differences in the biology of murine and human microglia (39), along with the lack of animal models for certain human CNS disorders, limit the applicability of these insights. Additionally, restricted access to human fetal brain tissue hinders direct investigation at cellular and molecular levels. As a result, *in-vitro* models have become essential tools for exploring the mechanisms of neurodevelopment.

This review critically evaluates the suitability of various *in-vitro* models for studying how variation in maternal-derived intrauterine factors affects fetal brain development, assessing their ability to replicate key physiological, genetic, and epigenetic conditions relevant to human development, and discussing their potential for translating findings from animal research into meaningful insights for human studies.

## 2 Critical analysis of *in-vitro* microglia models

### 2.1 Primary microglia cultures

Primary microglia cultures are derived directly from brain tissue, typically sourced from rodent embryos, neonates, or, less commonly, human fetal postmortem tissue (40, 41). The isolation process involves dissecting specific brain regions, dissociating tissues, and purifying microglia using techniques such as density gradient centrifugation, magnetic-activated cell sorting (MACS) (42), fluorescence-activated cell sorting (FACS) (43, 44), or orbital shaking (45). Because these cells are promptly isolated from fresh tissue sources, they retain essential microglial phenotypes and immune functions, allowing for physiological relevance in culture. This preservation of immune characteristics and cellular integrity makes primary microglia valuable for studying baseline microglial responses to various stimuli in a controlled environment.

Despite their physiological relevance, primary microglia cultures face limitations when used to model prenatal *in-utero* conditions. One major challenge is the impact of postmortem changes in brain cell structure. Studies (46–48) on postmortem brain tissue have shown that cell morphometry can be significantly altered depending on the postmortem interval. Cell death through oncotic necrosis, resulting from adenosine triphosphate depletion, leads to cell swelling, vacuolization, and loss of membrane integrity (46, 49). These changes can impair critical cellular functions, such as metabolic activity or signaling responsiveness (50), reducing the physiological relevance of isolated microglia for primary cultures. Additionally, fluid shifts and vacuolization during the postmortem interval can distort brain tissue structure (46), potentially inducing cellular stress responses that affect microglial behavior *in-vitro*.

In addition to degradation concerns, variability in prenatal exposures, such as maternal stress or infection history, introduces heterogeneity in microglial immune responsiveness and phenotypic characteristics, complicating reproducibility when facing limited availability of human fetal tissue. This further restricts the ability to obtain appropriately powered sample sizes and limits scalability for high-throughput studies. Moreover, practical and ethical constraints, alongside the labor-intensive nature of microglial isolation and culture, make primary microglia cultures less feasible for large-scale research.

In summary, while primary microglia cultures maintain physiological relevance and can capture certain baseline features of prenatal exposures, challenges related to postmortem cellular degradation, variability in intrauterine exposure, limited scalability, and ethical constraints reduce their suitability for comprehensively modeling mechanisms of fetal brain development.

### 2.2 Microglia cell lines

Microglia cell lines are widely used in neuroimmunological research due to their ease of handling, reproducibility, and scalability. These cell lines are typically derived from primary microglia obtained from brain or spinal cord tissues and are immortalized using viral transduction with oncogenes (51, 52) to ensure continuous proliferation. While this immortalization process allows for large-scale studies and consistent experimental outcomes, it introduces significant limitations that hinder their ability to accurately model the physiological, genetic, and epigenetic conditions necessary for understanding mechanisms of fetal brain development.

From a physiological perspective, microglia cell lines retain some core microglial functions, including adenosine triphosphate responsiveness, expression of macrophage/microglia marker, and basic phagocytic abilities (52). However, critical differences have been reported between primary microglia and cell lines in their responses to inflammatory stimuli (53–55). For example, the HMO6 cell line, developed by transducing embryonic microglia from telencephalon with a v-myc retroviral vector, showed limited ability to secrete a diverse range of inflammatory proteins upon exposure to lipopolysaccharide (LPS) or amyloid-β (52). Specifically, HMO6 cells showed limited secretion of interleukin (IL)-1β, IL-6, and macrophage inflammatory protein-1 α (MIP-α) compared to primary microglia (52). These functional deficits, likely attributable to the immortalization process, limit the utility of microglia cell lines for modeling the dynamic immune responses in the developing brain. Other cell lines, such as HMC3 (51) and C13NJ (56), are derived from the CHME-5 lineage, which has been suggested to originate from non-human sources, raising concerns about their validity for human-centered research (57).

In addition to physiological differences, the immortalization process may fundamentally alter the genetic and epigenetic landscape of microglia cell lines. Studies on cancer cell lines expressing SV40 T-antigen and oncogenic H-RAS demonstrate significant genetic and epigenetic changes over time, including disrupted tumor suppressor pathways, *de novo* DNA methylation at gene promoters, and transcriptional reprogramming (58). These changes lead to silencing of differentiation-associated genes, activation of cancer-associated signaling pathways, and acquisition of abnormal growth properties. While these findings are specific to cancer cells, the reliance on similar oncogenic transformation processes suggests that microglial cell lines may exhibit comparable alterations, including disrupted chromatin landscapes and gene expression, further reducing their suitability.

Such limitations compromise the utility of microglial cell lines for studying mechanisms of fetal programming, where precise epigenetic regulation is critical for understanding microglial roles in the developing brain. These shortcomings underscore the need for careful interpretation of results and the importance of combining microglia cell lines with other models, such as primary cultures or *in-vivo* systems, to better capture the complexity of prenatal brain development.

### 2.3 Stem cell-derived microglia

Stem cell technology offers promising opportunities for generating microglial cells in large quantities for *in-vitro* research. Microglia can be derived from embryonic stem cells (ESCs), harvested from the inner cell mass of a blastocyst, or induced pluripotent stem cells (iPSCs), reprogrammed from somatic cells (59, 60). Differentiating ESCs or iPSCs into microglia is a multi-step process, completed in around 30 days and often using co-culture systems with astrocytes to support microglial maturation (61, 62). However, stem cell-derived microglia often display immature phenotypes, limiting their physiological relevance for modeling prenatal environments. This is of particular importance because it has been previously shown that fetal microglia exposed to maternal inflammation, in a model of maternal immune activation, undergo accelerated maturation (63), a process that in this case stem cell-derived models may not fully replicate.

Further, the reprogramming and differentiation steps alter the cells’ epigenetic landscape (64, 65), which may impact their ability to accurately model *in-vivo* conditions. This poses a significant challenge for research on fetal programming, where maternal health and environmental exposures are associated with epigenetic modifications that affect long-term brain development. Consequently, stem cell-derived microglia may fail to capture these specific epigenetic changes, thereby limiting their suitability.

Despite these limitations, iPSC-derived microglia offer significant advantages, including patient-specific genetic backgrounds and precise experimental control. These models enable detailed studies of how genetic mutations, such as those associated with schizophrenia, influence microglial behavior, providing insights into neurodevelopmental disorders (66–68). However, operational challenges such as differentiation protocols are labor-intensive, costly, and require specialized conditions, limiting their scalability for high-throughput studies (60).

In summary, while iPSC-derived microglia are valuable tools for investigating molecular pathways and genetic variations, their occasional immature phenotypes, altered epigenetic profiles, and operational challenges limit their use in modeling the complex effects of maternal health and prenatal environmental factors on fetal brain development.

### 2.4 Three-dimensional brain organoids

Three-dimensional (3D) brain organoids, developed from iPSCs, represent a major advancement in modeling human brain development and disease (69). These self-organizing structures mimic key features of the human fetal brain, including cytoarchitecture, cellular diversity, and gene expression profiles, resembling the first and second trimesters of development (70–75). Organoids can generate region-specific structures, such as the forebrain, hippocampus, and midbrain, and advances in fused assembloid systems allow researchers to model connectivity between brain regions, an essential feature for studying neurodevelopmental disorders (76–80).

A major strength of 3D brain organoids is their high physiological relevance. Their spatial organization enables the investigation of neurodevelopmental processes, such as cell-cell interactions and responses to environmental influences, in a more lifelike setting. Incorporating iPSC-derived microglia enhances their complexity, allowing for the study of microglial functions, including synaptic pruning and neuroinflammation, within a neural context (81). This makes organoids particularly valuable for exploring how intrauterine environmental factors, like maternal stress, shape brain development.

However, 3D brain organoids face significant limitations. Generating them is technically demanding and resource-intensive, and there is considerable variability in size, cell composition, and maturation between batches, making standardization and reproducibility challenging (72, 82). Additionally, the lack of systemic features like vascularization limits their capacity to sustain long-term growth and fully replicate *in-vivo* conditions. Additionally, while organoids inherit the genetic background of donor iPSCs, the reprogramming process alters their epigenetic landscape (83, 84). This reduced epigenetic fidelity poses challenges for studying maternal influences that rely on specific epigenetic modifications, such as those induced by maternal stress or inflammation, which are critical to understanding mechanisms of fetal programming.

In summary, 3D brain organoids offer a high degree of physiological relevance and are powerful tools for modeling neurodevelopmental processes. However, their moderate epigenetic fidelity, technical complexity, and inability to replicate systemic features necessitate cautious interpretation of findings. To address these limitations, organoids should be used alongside other *in-vitro* or *in-vivo* models for a more comprehensive investigation of how maternal health influences the developing brain of the fetus.

### 2.5 Induced microglia-like cells from peripheral blood mononuclear cells

Peripheral blood mononuclear cell (PBMC)-induced microglia-like cells offer an accessible and scalable alternative for studying CNS-resident microglia. Derived from myeloid cells in PBMCs or cord blood mononuclear cells (CBMCs), these cells are differentiated into microglia-like cells within 10–14 days using growth factors such as macrophage colony-stimulating factor (M-CSF), granulocyte-macrophage colony-stimulating factor (GM-CSF), and IL-34 (85–89). This method is advantageous due to the ease of obtaining PBMCs through non-invasive blood draws and the availability of CBMCs collected at birth (90).

While PBMC-induced microglia-like cells express key microglial markers such as IBA1, CX3CR1, PU.1, P2RY12, and TMEM119, their hematopoietic origin fundamentally differs from CNS-resident microglia, which derive from yolk sac progenitors during embryogenesis (68, 90). This difference in lineage results in functional distinctions, as PBMC-derived cells resemble peripheral macrophages more closely than microglia, limiting their ability to model the unique roles of CNS microglia in prenatal brain development (68, 91, 92). Additionally, the epigenetic landscape of PBMC-derived cells is not CNS-specific. While cord blood-derived monocytes may retain some prenatal epigenetic marks, they likely reflect only the influence of maternal factors late in gestation and thus fail to capture the dynamic exposures and modifications occurring over time. This limits their relevance for studying the epigenetic mechanisms underlying fetal programming.

Despite these limitations, PBMC-induced microglia-like cells are highly practical for high-throughput studies due to their rapid and cost-effective generation. Their simple and scalable differentiation protocols make them accessible to labs with limited resources. However, variability in differentiation conditions can impact reproducibility, and the lack of interaction with other brain cell types, such as neurons and astrocytes, reduces their physiological relevance (93). While efforts to address these limitations, such as using simplified co-culture models to study synaptic interactions, have shown promise (68, 94), these cells fall short of comprehensively model neuroimmune interactions in the developing brain.

In summary, PBMC-induced microglia-like cells are valuable for basic microglial studies and large-scale investigations due to their accessibility and rapid generation. However, their distinct developmental origin and limited physiological and epigenetic fidelity restrict their application in studying maternal health impacts and fetal brain development. For a more complete understanding of prenatal programming, these cells should be used in combination with complementary models like iPSC-derived microglia or organoids.

### 2.6 Hofbauer cells

Hofbauer cells (HBCs), fetal macrophages originating from yolk sac progenitors, populate the chorionic villi of the placenta throughout pregnancy and share a developmental origin with CNS-resident microglia (95–97). Both cell types arise from the same progenitor lineage but migrate to distinct tissues, where they perform specialized immune functions. In the placenta, HBCs regulate immune responses, combat infections, and contribute to tissue remodeling, roles analogous to microglia in the developing brain (98, 99). This developmental and functional overlap makes HBCs a potential surrogate for studying mechanisms of fetal programming in humans.

One of the primary strengths of HBCs is their direct and continuous exposure to maternal conditions throughout gestation, allowing them to reflect intrauterine changes from the earliest stages of development. However, maternal factors not crossing placental or fetal barriers may limit HBCs’ fidelity. Previous research (95, 100) using differential gene expression (DEG) and Gene Ontology (GO) enrichment analyses has demonstrated significant alterations in HBC gene expression under maternal inflammatory conditions during pregnancy, affecting pathways related to immune signaling, metabolism, and cellular stress. Canonical pathway analyses have further shown shared responses between HBCs and fetal microglia, such as changes in glycolysis, oxidative stress responses, and inflammatory signaling under maternal obesity (95). These parallels highlight the potential of HBCs as proxies for investigating how maternal influences on fetal development.

Despite shared responses, important differences arise due to the distinct tissue environments in which HBCs and microglia reside. Microglia interact closely with neurons and CNS-specific cell types, contributing to neurodevelopmental processes like synaptic pruning and neural circuit refinement. In contrast, HBCs are influenced by placental functions, such as nutrient exchange, hormone production, and vascular development. For example, GO analyses reveal that mitochondrial metabolism and regulation of body fluid levels are uniquely enriched in HBCs, whereas microglia show enrichment in pathways related to neuron regulation and microtubule polymerization (95). These differences illustrate the tissue-specific adaptations of these macrophages, even under shared maternal health conditions.

HBCs are particularly well-suited for studying the effects of pre-conceptional maternal health, such as obesity, on fetal immune programming, as they are exposed to maternal factors from the earliest stages of development. However, they are less informative for maternal influences arising exclusively during pregnancy, as key developmental events, such as progenitor migration to the brain or placenta and blood-brain-barrier formation may have already occurred. This temporal limitation underscores the differences between HBC and fetal microglial responses to maternal health.

Practical challenges also restrict the broader applicability of HBCs. Their collection requires timely access to placental tissue at delivery, limiting scalability and accessibility. Furthermore, variability among placental samples, driven by maternal health factors such as infection and diet, complicates reproducibility and standardization. This heterogeneity poses challenges for studies requiring uniform cell populations or modeling precise biological processes.

In summary, Hofbauer cells are a valuable model for investigating how maternal health influences fetal brain development, particularly through mechanisms of immune programming shaped by pre-conceptional factors. Their high physiological relevance and potential to reflect early prenatal exposures make them well-suited for long-term developmental studies. However, limitations in scalability, reproducibility, and their ability to capture pregnancy-specific influences highlight the need for complementary models to achieve a comprehensive understanding of prenatal programming and its implications for fetal brain and immune development.

## 3 Discussion and conclusion

This review critically evaluates the suitability of various *in-vitro* models for studying the effects of maternal health conditions on fetal brain development, with a focus on their physiological relevance, genetic and epigenetic fidelity, and practical considerations such as scalability, reproducibility, and cost. Each model offers unique advantages and limitations, and none fully replicates the complex interactions between mother and child during pregnancy. Therefore, the choice of model should be guided by the specific research aims, the desired level of biological fidelity, and the available resources.

**Table 1** provides a comparative summary of these models, helping researchers select the most appropriate model for their study based on the criteria discussed. This resource highlights each model’s strengths and limitations, facilitating informed decisions in research planning.

**Table 1:** Practical considerations for *in-vitro* microglia models.

Model	Practical Guidance	Best Research Use	Time to Develop	Scalability	Technical Complexity	Costs	Epigenetic Fidelity	Physiological Relevance
Primary Microglia Cultures	Ensure consistent protocols to reduce variability; validate across batches and donors	Neurodevelopment, direct microglial responses	Medium	Low	Medium	Medium	Moderate	High
Microglia Cell Lines	Regularly monitor for changes in cell behavior; supplement with primary cultures or advanced models	High-throughput screening, basic immune response research	Short (days)	High	Low	Low	Low	Low
iPSC-Derived Microglia	Follow rigorous protocols; consider combining with other models; validate epigenetic markers	Neurodevelopment and neurodegenerative disorders	Long (weeks)	Medium	High	High	Low	Moderate
3D-Brain Organoids	Standardize culture conditions; account for variability	Complex neurodevelopment, cell-cell interaction studies	Long (weeks-months)	Low	High	High	Moderate	High
Induced Microglia-Like Cells from CBMCs	Optimize induction protocols; validate microglial markers and functional responses	General neuroinflammation, immune signaling pathways	Short (1-2 weeks)	High	Medium	Low	Low	Low
Hofbauer Cells	Collect placental tissue promptly; validate with CNS models; consider context specific use	Maternal-fetal immune interactions, placental influence on brain development	Short (days)	Medium	Medium	Medium	High	Moderate

iPSC, induced pluripotent stem cell; CMBCs, cord blood mononuclear blood cells; 3D, three dimensional; CNS, central nervous system.
